# The Value of Meat Extract

**Published:** 1871-11

**Authors:** 


### The Value of Meat Extract.

Meat, as an article of diet, owes its value partly to the mineral, partly to the organic compounds, albuminoid and oleaginous. The mineral substances probably undergo little change in the human body, and with the oleaginous we do not now concern ourselves; but the albuminoid having been ingested, are in the stomach reduced to a uniform substance, termed albuminose or peptone, by means of the acid and pepsin of the gastric juice. But straightway the process of degradation commences, and a multitude of new compounds, kreatin, kreatinine, leucin, etc., are formed; but the force evolved in the changes is the force manifested by the body—in other words, life implies these changes. Let it be noted now that in the *substance* of meat there is available material over and above what exists in its juice.

In the extract, substances which exist only in very small quantity in the meat are increased a hundred-fold, whilst the really important and nutritious substances are actually diminished. Animals have no constructive power that resides in vegetables. Albumen having ceased to be albumen, it can never be again converted into albumen in the animal economy; it can only proceed in its retrograde march to urea. This being so, it is evident that the nutritive value of meat in regard to its albuminoid constituents must be small. Some time ago Kimmerich astonished and, we may say, frightened the world by announcing that Liebig's extract was absolutely poisonous. In a recent article, however, *(Deutsches Klinick)*, he has considered the subject, pointing out the actual value of the extract. First, then, it causes a sensation of warmth in the stomach, it strengthens the heart's action and the circulation generally, acting as a stimulant rather than as an article of food. In its action it is allied to tea and coffee rather than meat. So also in cases of sickness will the solution of the extract, properly flavored, prove of value as a stimulant, in the same way that a glass of wine will enable a man, immediately after he has taken it, to do what he was not able to do before. But, however useful in debility, it is now well known that wine is not food, and so also with meat extract. The real use of the meat extract, as an article of diet, is to give taste and relish to a mass of nutritive but insipid food. A patient will swallow large quantities of this extract, thinking he is imbibing nutriment proportional to the quantity of meat used in its preparation, while in truth he may swallow several ounces of it daily and yet be starving. A bone boiled with it for a time is a great improvement; thickening it with *corn-meal,* not starch, makes it of very great value.—*Medical Times and Gazette.—New Remedies.*

